# Malignancy-mimicking Kayexalate-associated extraintestinal mass and literature review

**DOI:** 10.1007/s12328-026-02371-8

**Published:** 2026-05-26

**Authors:** Jenna Magri, Chadi Hajar, Thomas Curran, David Lewin

**Affiliations:** Medical University of South Carolina, 171 Ashley Avenue, Charleston, SC 29425 USA

**Keywords:** Kayexalate crystals, Hyperkalemia, Malignancy-mimicking mass

## Abstract

The gastrointestinal side effects of Kayexalate administration are well-established, and despite its discontinued use in the United States, it is still important to consider in the clinical and pathologic differential, given the vast time range between administration and clinical presentation. One less common, though significant, complication of Kayexalate use is the formation of a malignancy-mimicking mass or pseudotumor. This presentation has been reported several times both within and outside of the gastrointestinal tract. Here we describe the case of a 65-year-old female presenting with a mass medial to the ascending colon with extension to the abdominal wall and adjacent bowel concerning for metastatic malignancy. Additionally, we review the current literature on Kayexalate associated pseudotumors and malignancy-mimicking masses.

## Introduction

Kayexalate (sodium polystyrene sulfonate) is a cation-exchange resin used in the treatment of hyperkalemia. It has well-recognized gastrointestinal adverse effects, which include mucosal inflammation, ulceration, and fatal bowel necrosis. It is now discontinued in the United States, though generic forms of sodium polystyrene sulfonate still exist along with other newer potassium lowering agents [1–5]. Of note, Kayexalate historically has been administered with and without sorbitol; both substances, alone and in combination, have been implicated as causes of gastrointestinal injury, though the mechanism is not well understood [1–2, 6]. Histologically, the affected tissue will show evidence of inflammation, ulceration, necrosis, and/or perforation, depending on the severity of disease, with classic “fish-scale” like basophilic refractile Kayexalate crystals visualized within the tissue [7].

Multiple case reports and reviews have exemplified the potential gastrointestinal injury associated with Kayexalate use, including potentially lethal ulceration, perforation, and necrosis of not just the small and large bowels but also the esophagus [1–2, 4, 8–12]. The reported case of lethal Kayexalate associated gastrointestinal perforation resulted in septic shock and death in a ventilator dependent trauma patient with tracheostomy and gastrostomy tube. At autopsy, Kayexalate crystals were identified as expected in the gastrointestinal system, including gastroesophageal junction, stomach, small and large bowels, but also systemically within or in peri-organ adipose tissue of the gallbladder, appendix, pancreas, spleen, bladder, thyroid gland, skeletal muscle, and subcarinal lymph node with no definitive mechanism for distant spread [12]. This case demonstrates the intra-intestinal and extraintestinal complications of Kayexalate use and subsequent crystal deposition. One very rare complication of Kayexalate use, described only a few times in the literature, is the formation of a malignancy mimicking mass or pseudotumor. These reports describe masses within the gastrointestinal tract, including the esophagus [13] and colon [14–15], and outside of the gastrointestinal tract, including the perihepatic space [16], pre-sacral region [17], and pelvis [5]. Here we describe the case of a 65-year-old female presenting with a mass medial to the ascending colon with extension to the abdominal wall and adjacent bowel concerning for metastatic malignancy.

### Case report

**Fig. 1:**
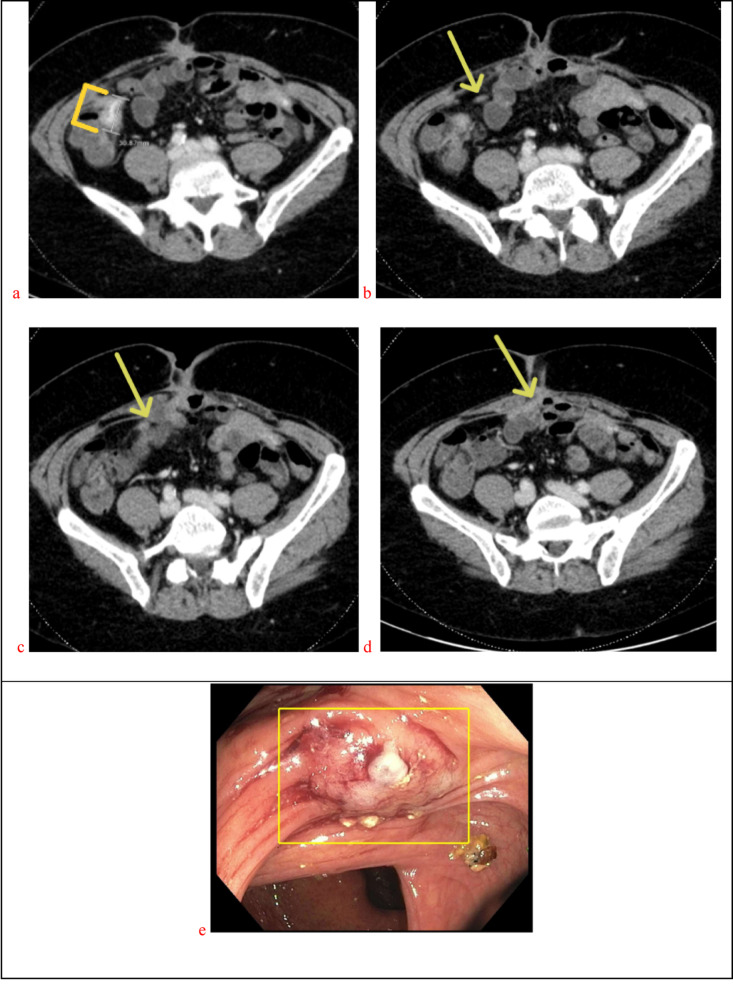
Preoperative radiographic and colonoscopy images. **a–d**. Computed tomography (CT) with contrast shows a hyperattenuating 3.1 cm mass-like lesion (bracket) medial to the ascending colon with hyperattenuating linear areas extending from the mass toward the anterior abdominal wall and extending to the midline and left anterior abdomen (arrows).** e**. Colonoscopy shows a 2.0 cm sessile and ulcerated non-obstructing mass within the ascending colon

A 65-year-old female with past medical history of bipolar I disorder, hypertension, hypercholesterolemia, hysterectomy, oophorectomy, and appendectomy with a long history of abdominal pain and recurrent bowel obstructions (conservatively treated) underwent exploratory laparotomy and lysis of adhesions for sepsis attributed to a small bowel obstruction complicated by enterotomy and intra-abdominal abscesses in 2019. She was transferred to a higher level of care due to recurrent small bowel obstruction and a complicated recovery course. After transfer, computed tomography (CT) of the abdomen and pelvis with contrast demonstrated a 2.8 cm hyperattenuating mass-like structure medial to the ascending colon. Repeat CT in 2022 showed an increase in mass size, to 3.1 cm, with extraintestinal extension to the anterior abdominal wall and adjacent small bowel loops concerning for metastatic malignancy (Fig. 1). Colonoscopy showed a 2.0 cm sessile and ulcerated non-obstructing mass within the ascending colon which was biopsied to show ulceration of the colonic mucosa with associated crystals consistent with Kayexalate crystals (Fig. 1).

**Fig. 2:**
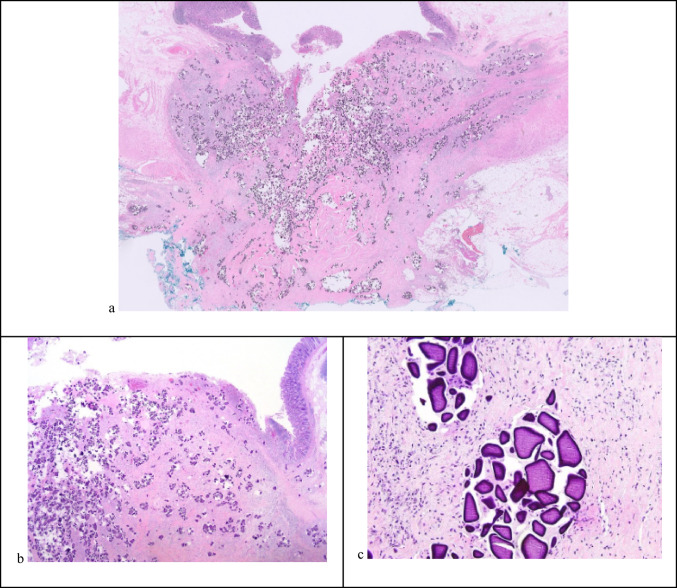
Histopathologic images. **a**. Full thickness penetration of the ascending colon by Kayexalate crystals, extending from the ulcerated mucosal surface through to the blue-inked serosa, with surrounding histiocytic inflammatory infiltrate and fibrosis, distinct from the adjacent relatively normal appearing colon on both sides of the lesion (H&E, 10x)** b**. Normal appearing uninvolved colonic mucosa on the right transitions to focally ulcerated mucosa with diffuse infiltration of Kayexalate crystals into the submucosal tissue (H&E, 20x)** c**. Kayexalate crystals imbedded in the abdominal wall have a basophilic, polygonal, fish-scale like appearance and are surrounded by a histiocytic inflammatory infiltrate and fibrosis (H&E, 200x)

The patient was then lost to follow up until 2024 when she presented with persistent chronic abdominal pain, intermittent bloody stool, and alternating constipation and diarrhea. Repeat CT imaging at that time showed a 2.6 cm mass medial to the ascending colon with extension to the anterior abdominal wall and adjacent small bowel loops concerning for malignancy. The patient underwent a diagnostic laparoscopy with lysis of adhesions, open right hemicolectomy, small bowel resection, and debridement of the abdominal wall. A portion of abdominal wall mass tissue sent intraoperatively for frozen evaluation due to concern for potential malignancy was interpreted as fibrosis, and histiocytic infiltrates with diffuse crystal deposits consistent with Kayexalate. No obvious malignancy was identified. Evaluation of the permanent histologic slides confirmed the findings of the abdominal wall mass, showing fibrosis with diffuse Kayexalate crystals and histiocytic infiltrate without malignant cells. In addition, focal ulceration was noted in the ascending colon with Kayexalate crystal deposition and histiocytic infiltrate (Fig. 2). Sectioning through the ulcerated area demonstrated diffuse deposition of Kayexalate crystals within the bowel wall with focal perforation and serositis. Detached fragments of Kayexalate crystals were also noted in a portion of the ileum. A presumptive diagnosis of ulcerated large bowel with perforation and serositis associated with foreign body crystal deposition consistent with Kayexalate was made, though no administration history could be confirmed due to the limited availability of prior outside medical records.

**Fig. 3:**
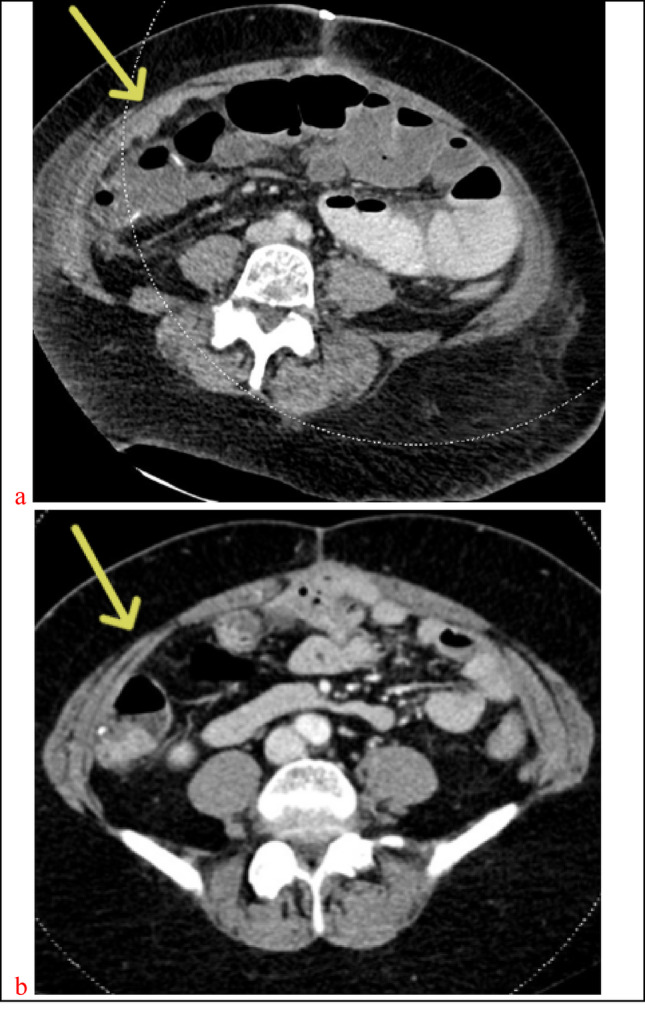
Postoperative radiographic images. **a**. Immediate post-operative Computed Tomography (CT) imaging shows postsurgical changes from the right hemicolectomy and small bowel resection with hyperdense thickening of the peritoneum in the right lower quadrant (arrow), which may be postsurgical or represent residual disease.** b**. CT imaging 15 months post-op shows the same postsurgical changes from right hemicolectomy and small bowel resection without peritoneal thickening

The patient’s postoperative course was complicated by nausea, vomiting, bloody bowel movements and anemia requiring red blood cell transfusion, and ileus resolved via nasogastric tube before being discharged to home health after twenty-seven days. Immediate postoperative CT imaging showed postsurgical changes from right hemicolectomy and small bowel resection with hyperdense thickening of the peritoneum in the right lower quadrant, which was described as potentially representing postsurgical change or residual disease (Fig. 3). On subsequent imaging at 15 months post-op, the peritoneal thickening was resolved with only postsurgical changes noted (Fig. 3). The patient continued to seek treatment with multiple hospital admissions for persistent intractable abdominal pain, nausea, vomiting, diarrhea, and dysphagia, prompting cholecystectomy approximately 1 year post-op. An esophagogastroduodenoscopy (EGD) over 1 year post-op showed ulceration of the esophageal mucosa with histologic examination of the esophagus, stomach, and duodenum revealing benign mucosa with erosion in the esophagus but no Kayexalate crystals. The patient continues to experience chronic intractable abdominal pain 1.5 years post-op with no further colorectal pathology on subsequent imaging. 

## Discussion with literature review

This case exhibits a rare complication of Kayexalate use presenting as a mass mimicking malignancy. Previous reports of similar malignancy-mimicking Kayexalate related cases vary in location, clinical presentation, size, time between Kayexalate use and presentation, and treatment course (Table 1). The intra-gastrointestinal cases were localized to either the ileocecal valve within the cecum [14–15] or the gastroesophageal junction (GEJ) of the esophagus [1–2], ranging in size from 3 to 5 cm. The extraintestinal cases were variable, localized to the perihepatic space adjacent to a colostomy site [16], pre-sacral region [17], and the pelvis between the bladder and rectum [5], ranging in size from 6.7 up to 11.0 cm. Radiographically, the different mass-like lesions were described using terms such as “heterogenous” [5, 16–17], “ill-defined” [14] or “indeterminate” [17], with some exhibiting “peripheral enhancement” and “central necrosis” [5] and others having “localized inflammatory changes” [14]. Despite the variable descriptors, all six cases appear to be a single relatively contained mass with no evidence of potential extension or metastasis based on the available information. Endoscopic examination was performed in four of six cases reviewed here, with observable changes found only in the three intra-gastrointestinal cases, including erythema, ulceration, and mass formation [14, 15], one of which was described as appearing similar to esophageal carcinoma [13].

The treatment course for each case was also variable, though intra-gastrointestinal cases tended towards more conservative management compared to extra-intestinal cases. Aside from obtaining a biopsy, the intra-gastrointestinal cases were treated with surveillance and non-surgical management [13, 15] as well as aborted subtotal colectomy after confirming the absence of carcinoma via intraoperative consultation [14]. The treatment courses of the extra-intestinal cases were more limited in description, with a greater focus on the diagnostic methods and less on the ensuing treatment. These treatments ranged from endoscopic ultrasound guided fine needle aspiration with no apparent subsequent intervention [5] to laparotomy for surgical evacuation of a presumed hematoma and/or mass excision [16–17]. Overall, it appears that cases without confirmed diagnosis excluding malignancy and those that are located outside of the intestinal tract are more likely to be treated with more aggressive surgical intervention. Of note, none of the cases described in the literature indicated a concern for metastatic disease or extension into adjacent structures as in our case.

Finally, the reported cases of a mass-like complication of Kayexalate use presented with variable relationship between Kayexalate administration and presentation, including length and duration of use. The most acute presentation consisted of mass formation within the cecum after only two doses of Kayexalate during the same admission [15]. Alternatively, the longest time between Kayexalate use and identification of mass-like complications was over 10 years post-administration [5]. Other reviews of Kayexalate related gastrointestinal injury also show wide variations in length and duration of Kayexalate use and time between use and complication development or presentation, consistent with this subset of mass-like complications [1–2].

In our case, an ulcerated mass was visually identified on colonoscopy with radiographic evidence of an extraintestinal mass medial to the ascending colon with extension, mimicking potential metastasis, to the abdominal wall and adjacent bowel. During surgical evaluation, the concern for malignancy was strong enough to submit tissue for an intraoperative frozen evaluation to best direct treatment. The treatment was extensive, with removal of a portion of large and small bowel and a portion of abdominal wall, reflective of the extensive involvement of the disease process as well as the patient’s preference for surgical removal in an attempt to alleviate chronic abdominal pain and gastrointestinal symptoms. Our patient’s clinical presentation was similar to previous reports of mass-like Kayexalate related complications with abdominal pain and altered bowel habits, and her age of 65 is close to the mean age of 65.8 years for the six other reported cases (Table 1).

**Table 1:** Reported cases of pseudotumor/mass formation associated with Kayexalate use

Author	Gender	Age	Clinical presentation	Location	Endoscopy/ colonoscopy findings	Radiographic findings	Kayexalate use history	Treatment
Extraintestinal cases								
Romano et al. [16]	Female	62	Abdominal pain	Extraintestinal Perihepatic space adjacent to colostomy site	–	11.0 cm heterogenous mass consistent with hematoma	Oral, 3 years of chronic Kayexalate use prior to presentation	Surgical evacuation of “hematoma”, with subsequent biopsy sampling of persistent mass
Goh et al. [17]	Female	59	Asymptomatic	Extraintestinal Pre-sacral	Unremarkable	Indeterminate pre-sacral heterogenous solid mass	Oral, 2 three-day courses of Kayexalate 1 year and 12 years prior to presentation	Diagnostic laparotomy and mass excision
Banihashemi et al. [5]	Male	55	–	Extraintestinal Pelvic, between the bladder and rectum	–	6.7 cm heterogenous mass between bladder and rectum with peripheral enhancement and central necrosis	Most recent Kayexalate use over 10 years prior to presentation *(route of administration not provided)*	Endoscopic Ultrasound-Guided Fine-Needle Aspiration; no further intervention described)
Intra-gastrointestinal cases								
Bui et al. [14]	Female	70	Change in bowel habits (constipation & loose stool)	Intra-intestinal Cecum at the ileocecal valve	Gastritis, duodenitis, mass at ileocecal valve, internal hemorrhoids	Ill-defined 5 cm cecal mass with localized inflammatory changes	Oral, 1 year of chronic Kayexalate use (15 g twice daily)	Aborted subtotal colectomy after intraoperative consultation showed no carcinoma
Chatila et al. [15]	Male	66	Right flank pain, hyperkalemia, acute kidney injury	Intra-intestinal Cecum at the ileocecal valve	Large mass adjacent to the ileocecal valve	3 cm mass in the cecum	2 doses at presentation *(route of administration not provided)*	Repeat colonoscopy with biopsy 8 days after initial colonoscopy; no further intervention described
Chavez et al. [13]	Male	83	Cough, emesis, melena, dysphagia, weight loss	Intra-esophageal Gastroesophageal junction (GEJ)	Esophageal mucosa with erythema, congestion, deep ulcerations, and partially obstructive mass in GEJ	Dilated esophagus with air fluid level, achalasia-like appearance, and mass near the GEJ	1 week of Kayexalate 6 weeks prior to presentation *(route of administration not provided)*	Proton pump inhibitor with supportive non-surgical management and surveillance

It is important to acknowledge that in our case, the diagnosis was made presumptively, as it is unclear to the clinicians and surgeons when exactly the patient received sodium polystyrene sulfonate during her complicated medical history. However, with the diagnoses of hyperkalemia and acute kidney injury listed in transfer paperwork in 2019 without documented evidence of chronic kidney disease or another disease state and/or medication that would suggest prior long term hyperkalemia requiring treatment, it is presumed that the patient received Kayexalate for a brief duration during the three weeks she was admitted for sepsis related bowel obstruction and perforation prior to transferring facilities; though administration(s) prior to 2019 cannot be excluded. There is no indication that the medication dosage would have been outside of routine guidelines and given the identification of crystals within the small bowel in addition to the large bowel, the administration route is favored to be oral instead of rectal via enema. Given the limited available records, direct comparison with previously reported cases, particularly regarding the relationship between Kayexalate administration and clinical presentation, remains constrained.

Despite the discontinuation of Kayexalate production in the United States, it is still crucial for clinicians, surgeons, and pathologists to recognize and accurately diagnose the gastrointestinal complications of Kayexalate, given the widely variable time interval between Kayexalate use and presentation of complications, up to 10 years, and the continued availability of generic sodium polystyrene sulfonate. In patients with any history of Kayexalate or sodium polystyrene sulfonate use presenting with potential gastrointestinal or extraintestinal masses, the possibility of Kayexalate related mass formation should be considered in the differential and evaluated early in management to avoid potentially unnecessary extreme surgical or medical management.

This case highlights the critical role of microscopic examination in identifying clinically significant medication-related adverse events, particularly in patients with limited medication history and prior hospitalization documentation. Importantly, this case is also a reminder that kayexalate-associated gastrointestinal injury may represent a delayed manifestation, occurring years after potential administration, and highlights the need to consider prior resin exposure in the differential diagnosis of unexplained bowel perforation even when remote or undocumented.
